# Supplementary material for: Primary visual cortex straightens natural video trajectories
Source: Nat Commun. 2021 Oct 13;12:5982. doi: 10.1038/s41467-021-25939-z (PMC8514453; doi:10.1038/s41467-021-25939-z)
Supplement: Supplementary file 1 — Supplementary Information [file 41467_2021_25939_MOESM1_ESM.pdf]

Supplementary Information:

Primary visual cortex straightens natural video trajectories

Olivier J. Hénaff*, Yoon Bai*, Julie Charlton, Ian Nauhaus, Eero P. Simoncelli, Robbe L. T. Goris

Inferring unbiased estimates of neural curvature

We evaluated our ability to recover known curvature values in simulation, using two estimators. The first estimated the most likely neural trajectory (using a trial average) and computed its curvature. Because of the inherent uncertainty in estimating neural firing rates from a realistic number of experimental trials, the resulting curvature estimates proved to be severely biased (Supplementary Figure 1a). Indeed, even in modestly-sized neural populations, estimation noise pushes trial-averaged population vectors towards orthogonality. Mutually orthogonal vectors with the same norm form an equilateral triangle, whose inner angles span 60° and outer angles (or curvature) 120°, explaining why the recovered curvature tends towards this value.

The second estimator, used in the analyses of this paper, infers not just the single most likely neural trajectory, but a distribution over plausible neural trajectories, using variational Bayesian inference (see Methods). By properly accounting for the uncertainty in neural firing rates (i.e. estimating and marginalizing over them), this procedure yields largely unbiased neural curvature estimates (Supplementary Figure 1b).

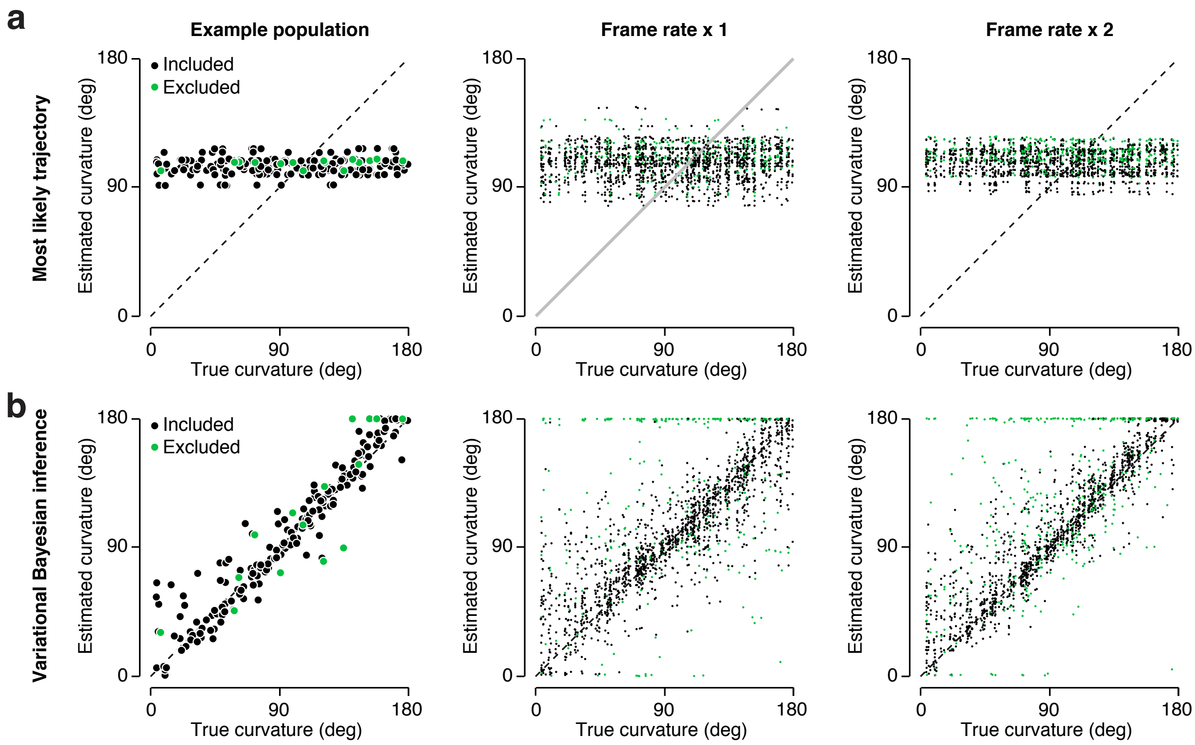

Supplementary Figure 1 Recovery analysis to evaluate neural curvature estimation procedures. (a–b) For each population, we simulated 200 neural trajectories whose curvature was randomly sampled from 0° to 180°, and whose other parameters (path length, dimensionality, etc.) were matched to those of the empirical data sets. Each of the 20 empirically estimated natural video trajectories was used 10 times in this analysis. We compared two estimation procedures: (a) the curvature estimate of the single most likely neural trajectory, and (b) the average curvature estimate across a distribution of plausible neural trajectories. Left column: one example population (population 4 from Figure 3), using trajectories at the fine temporal scale. Middle and right columns: all neural populations at the coarse (middle) and fine (right) temporal scales. Black points indicate data sets that meet the inclusion criteria, green points indicate data sets that fail to meet these criteria.

Evaluating the fit of inferred neural trajectories

Having inferred a distribution of "plausible" neural trajectories, we verified that these trajectories correctly described the basic features of the data: response mean, variance and covariance. To do so, we selected the neural trajectory which best captured the distribution over neural distances and curvatures (see Methods), and used it to generate predictions regarding these statistics. We then compared them to the empirically observed spike count mean, variance and covariance (Supplementary Figure 2).

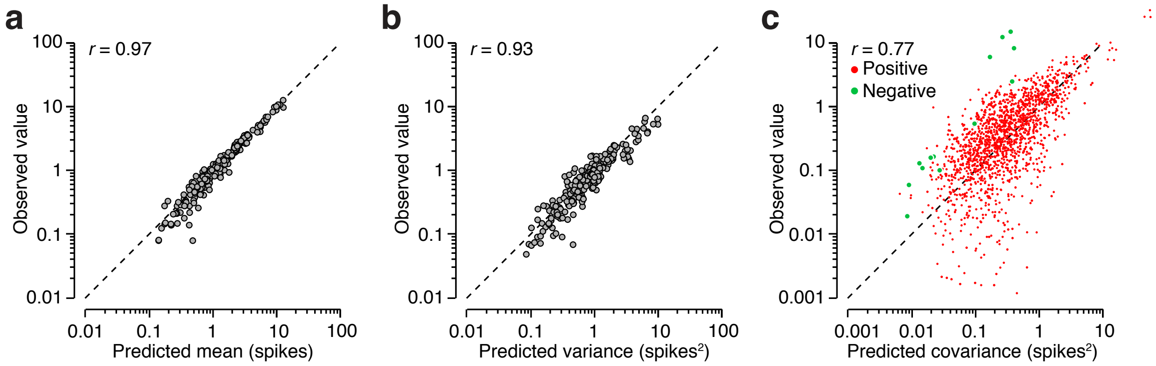

Supplementary Figure 2 Evaluating the goodness-of-fit of the descriptive response model. (a–c) Empirically observed response mean (a), variance (b), and covariance (c) as a function of the model's predicted values for the example dataset of Figure 2 ("Population 7", "Movie 7", "Frame rate x 1"). Each point illustrates the value for a single unit (a–b) or for a pair of units (c) in response to a single frame of the six-frame movie. (c) Positive-valued response covariance values are shown in red, negative ones in green. We used the Pearson correlation between log-transformed predicted and measured values as measure of goodness-of-fit.

Image sequences used in the main experiment

We presented sequences of images taken from 10 natural and 10 unnatural video clips. In Supplementary Figure 3 we show the full sequence of frames (at "frame rate × 1", i.e. 6 frames) for the 4 sequences eliciting maximal and minimal straightening and entangling: 'prairie', 'smile', 'carnegie dam', and 'bees' (see Methods).

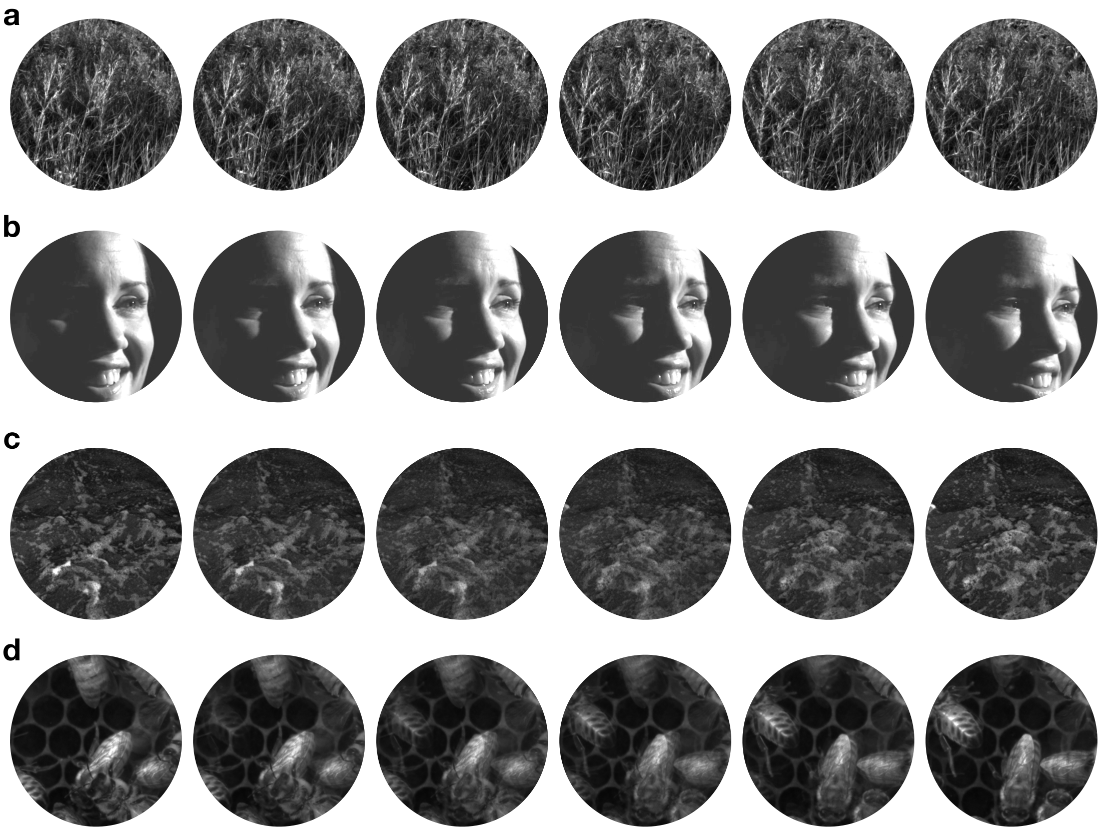

Supplementary Figure 3 Sequences eliciting strongest and weakest straightening (Figure 3C) and entangling effects (Figure 5C). (a) The strongest straightening effect was elicited by a natural sequence of a prairie, primarily consisting of shrubs and grass textures (zoom x 1, movie #20 in Figure 3C). (b) A sequence of a rotating face was the least straightened natural sequence (zoom x 2, movie #1 in Figure 3C). (c) Entangling was weakest for an unnatural sequence of waves ("Carnegie-dam", zoom x 2, movie #15 in Figure 5C). (d) Entangling was strongest for an unnatural sequence showing bees moving in a hive (zoom x 2, movie #4 in Figure 5C).

Assessing the impact of neural curvature debiasing

In Supplementary Figure 1a we found that failing to account for neural variability and covariability led to severe estimation bias. However even when fully taking these properties into account, neural curvature estimates may contain a residual bias, particularly for sequences with very low curvature (Supplementary Figure 1b). We therefore compare our neural curvature estimates to an explicit null-model which contains the same bias as neural curvature, and report "relative curvature" (i.e. the difference between neural curvature and that of the null-model) as a debiased statistic.

While this debiasing strategy is essential for assessing whether artificial sequences (which have close to zero pixel-domain curvature) are entangled, we asked whether it may have impacted our assessment of whether natural sequences are straightened. We therefore compared our unbiased estimates to the biased ones we would have obtained had we compared neural curvature directly to the pixel-domain curvature, rather than to that of the null-distribution (Supplementary Figure 4). In this case, the bias in our estimates would only increase the apparent straightening (from a median of 9.9^{o} to 10.2^{o}). Therefore, while we consider that comparing to an explicit null model is the more principled approach, our conclusions are robust to this choice.

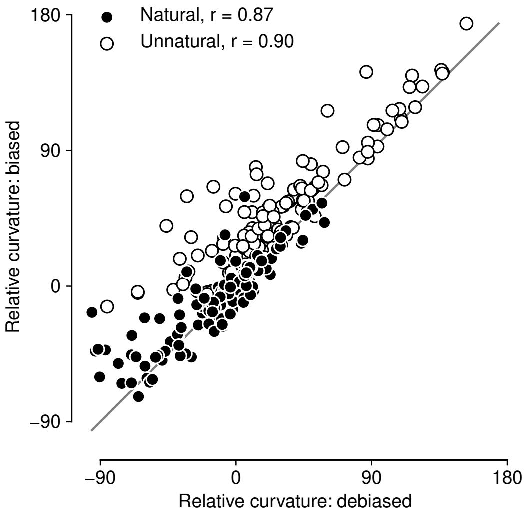

Supplementary Figure 4 Comparison of relative curvature estimates obtained using our debiasing strategy, with biased estimates which compare estimated neural curvature directly to pixel-domain curvature. In order to debias these estimates, we instead compare to the average curvature of a null-model whose true curvature is equal to the pixel-domain curvature, but whose estimates inherit the same bias as the neural curvature estimates. Median relative curvature, natural sequences: –9.9° (debiased), –10.2° (biased); unnatural sequences: 24.4° (debiased), 48.8° (biased).

Analysis of pixel-domain curvature across spatiotemporal scales

In Figure 6 we ask whether videos which have the same probability of occurring in the real world are equally straightened by neural populations. To test this, our set of image sequences is composed of two spatial scales ("zoom $\times$ 1" and "zoom $\times$ 2") and two temporal scales ("frame rate $\times$ 1" and "frame rate $\times$ 2"). Natural image sequences are approximately invariant to both spatial and temporal scaling. We verify this for the scales we consider by comparing the pixel-domain curvature across both spatial and temporal scales (Supplementary Figure 5). Consistently with the argument that the statistics of natural image sequences are invariant across the spatiotemporal scales we consider, we find their curvature to be highly conserved across both spatial (r = 0.96) and temporal (r = 0.91) scales. The pixel-domain curvature of unnatural sequences is zero by construction, with only slight deviations due to the quantization of individual frames required for presentation on a monitor. These deviations are also highly consistent across scales.

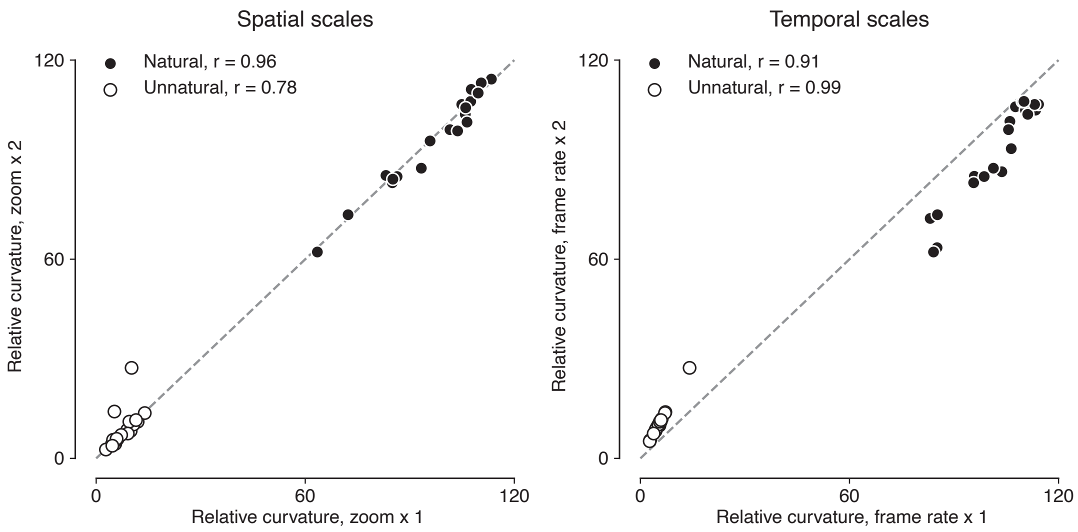

Supplementary Figure 5 Comparison of pixel-domain curvature across spatial and temporal scales. We use Spearman correlation to quantify the relationship across scales.

Further analysis of the dependence of straightening on firing rate

In Figure 6c we found that sequences which elicit higher firing rates are also straightened more by neural populations in V1. We asked whether this could be an artifact of our estimation, or whether it is a genuine physiological effect. We therefore decomposed the 'relative curvature' value in Figure 6c into its two constituents: straightening by neural populations (Supplementary Figure 6a), and straightening by the null-model (i.e. the estimation bias, Supplementary Figure 6b). The estimation bias exhibits no systematic dependency on firing rate, indicating that the systematic increase in straightening with firing rate is indeed a real physiological effect. Why do better driven populations straighten natural movies more strongly? One possibility is that movies that contain cells' preferred features engage non-linearities of V1 circuits in a unique manner, and that these non-linearities in turn act to straighten the temporal response trajectory. There is some evidence for the existence of such specialized non-linear mechanisms in complex cells, but not in simple cells (Felsen, Touryan, Han, and Dan, 2005 – PLoS Biology: Contrast-sensitivity to Visual Features in Natural Scenes).

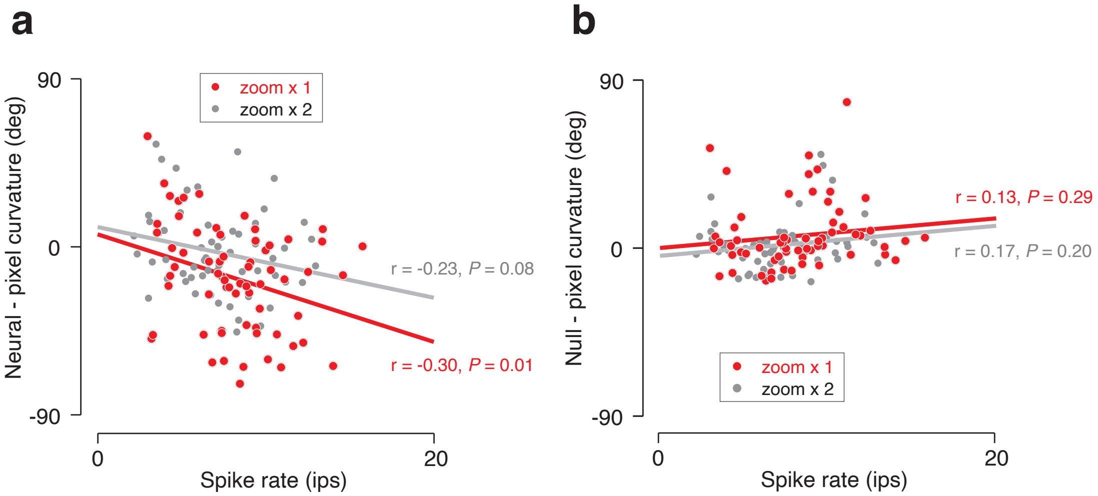

Supplementary Figure 6 Curvature values as a function of average firing rate for all natural movies, across two spatial scales. We dissected Figure 6C's 'relative curvature' into its two elements: (a) neural - pixel curvature, and (b) null model - pixel curvature, as a function of spike rates (i.e. the difference between (a) and (b) produces Figure 6C). Each point illustrates a dataset, following the same color code as in Figure 6C (red: zoom x 1, grey: zoom x 2). Regression lines are plotted in bold lines and annotated with Pearson's correlation coefficients and their associated p-values (two-tailed test).

Assessing the impact of neural variability and covariability on neural curvature estimation

Neural variability enters into the estimation of neural curvature twice. First, given a set of observed spike counts, neural variability determines the corresponding distribution of plausible neural trajectories. In Supplementary Figure 1 we found that properly estimating this distribution was essential for inferring reliable curvature estimates. Consistently with this, we found that ignoring neural covariability (noise correlations) when estimating this distribution greatly impaired our curvature estimates (Supplementary Figure 7a). This was also the case when considering neural trajectories at a finer temporal scale (Figure 1g). Ignoring both neural variability and covariability further impairs curvature estimates (Supplementary Figure 7b).

Neural variability also enters into the estimation of discriminability of pairs of response distributions (Figure 1e), which are used to obtain curvature estimates of each trajectory. In practice, however, we find that ignoring neural covariability in the definition of neural curvature has little impact, justifying the use of a simplified estimation procedure (Supplementary Figure 7c, see Methods). Ignoring both neural variability and covariability in this definition (i.e. directly measuring the curvature of firing rate trajectories) yielded very similar estimates (Supplementary Figure 7d).

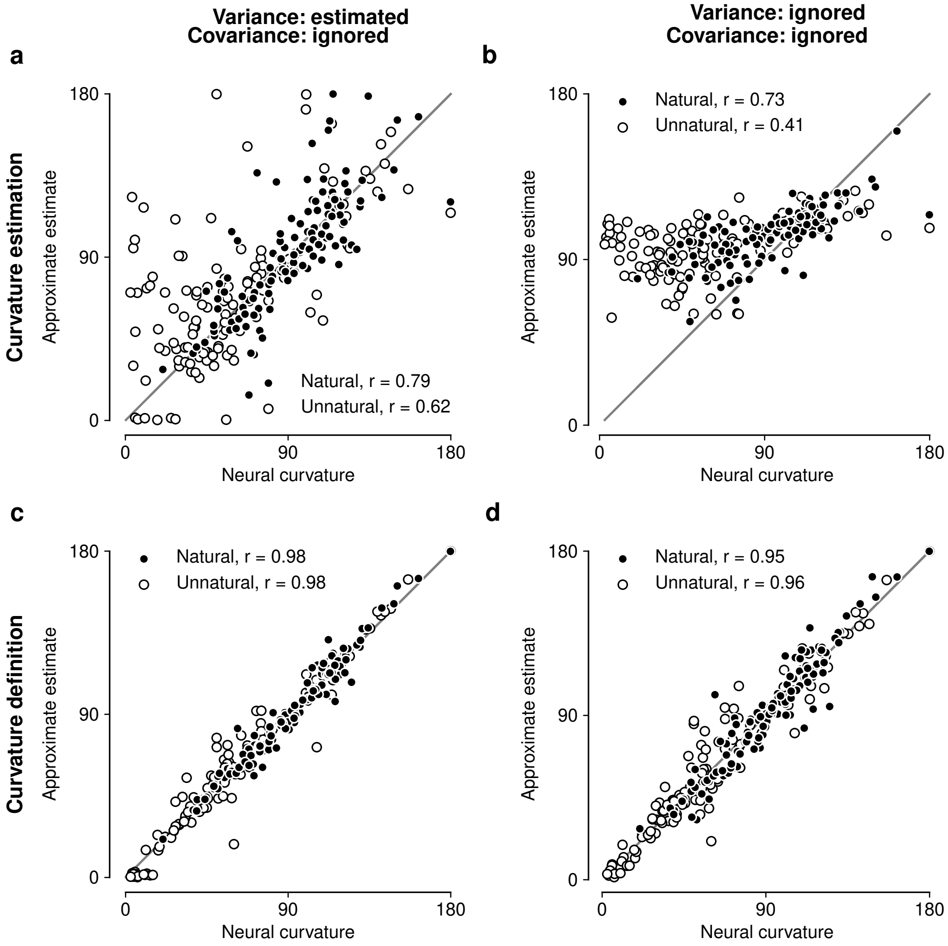

Supplementary Figure 7 Assessing the impact of neural variability and covariability on curvature estimates from the set of V1 population trajectories studied in this paper (coarse temporal scale). Our most comprehensive estimate defines curvature in terms of discriminability (which takes neural variability and covariability into account - see "Numerical curvature calculation" in Methods), and estimates it from a distribution of neural trajectories (which also takes them into account). We compare these full estimates (horizontal axis) to those obtained with four simpler approximations (vertical axis): (a) An impoverished descriptive model which ignores noise correlations when inferring the distribution of neural trajectories (see "pseudo-population estimates" in Methods). This method fails to provide reliable estimates of neural curvature. (b) Additionally ignoring neural variability when inferring this distribution further degrades the estimates. (c) Accounting for neural variability and covariability in the trajectory distribution, while ignoring covariability in the definition of neural curvature produces relatively small changes in estimates (see "population-based estimates" in Methods). (d) Additionally ignoring neural variability in the definition of neural curvature similarly has little effect.

Assessing the impact of a model-based inclusion criterion

After fitting our descriptive model to the spiking activity associated with a particular image sequence, we first assess the quality of the model fit and only proceed to measure neural curvature if the quality of this fit is high enough. As such we only consider curvature estimates which accurately reflect the data they were inferred from.

Nevertheless, we asked whether our results could be biased by the use of the inclusion criterion. We compared the distribution of straightening values obtained from sequences that met our inclusion criterion, and those that did not. They can largely be described as following the same distribution (Supplementary Figure 8). Comparing aggregate statistics, the effects we report are, if anything, emphasized if we include all sequences (median relative curvature, natural sequences: -9.9^{o} with included sequences, -10.1^{o} with all sequences; unnatural sequences: $+24.4^{o}$ with included sequences, $+27.1^{o}$ with all). We conclude that our results are not a simple byproduct of the inclusion criterion.

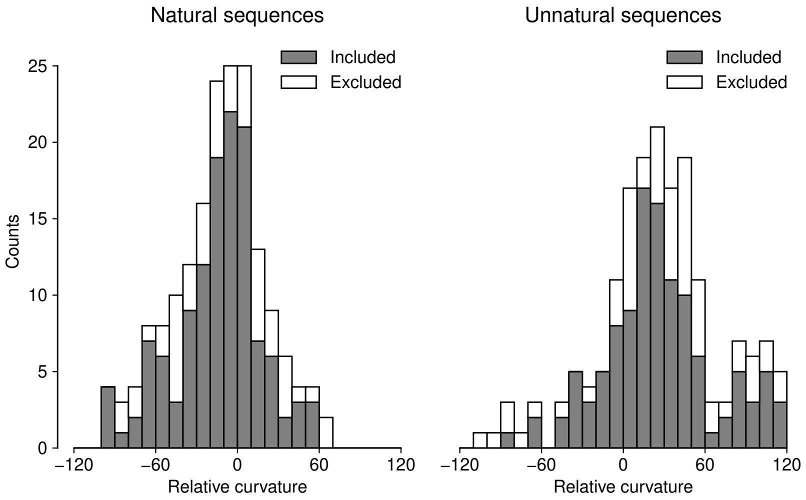

Supplementary Figure 8 Comparison of relative curvature of trajectories which meet our inclusion criterion (grey) and those that do not (white). The inclusion criterion only retains trajectories which provide an adequate fit to the observed spike counts, and whose length is above a certain threshold (allowing reliable curvature estimation).
