# Supplementary material for: Primary visual cortex straightens natural video trajectories
Source: Nat Commun. 2021 Oct 13;12:5982. doi: 10.1038/s41467-021-25939-z (PMC8514453; doi:10.1038/s41467-021-25939-z)
Supplement: Supplementary file 3 — Reporting Summary [file 41467_2021_25939_MOESM3_ESM.pdf]

nature portfolio

Corresponding author(s): Olivier Hénaff & Robbe Goris

Last updated by author(s): 8/8/2021

Reporting Summary

Nature Portfolio wishes to improve the reproducibility of the work that we publish. This form provides structure for consistency and transparency in reporting. For further information on Nature Portfolio policies, see our Editorial Policies and the Editorial Policy Checklist.

Statistics

For all statistical analyses, confirm that the following items are present in the figure legend, table legend, main text, or Methods section.

n/a	Confirmed	
☐	☒	The exact sample size (n) for each experimental group/condition, given as a discrete number and unit of measurement
☐	☒	A statement on whether measurements were taken from distinct samples or whether the same sample was measured repeatedly
☐	☒	The statistical test(s) used AND whether they are one- or two-sided<br>*Only common tests should be described solely by name; describe more complex techniques in the Methods section.*
☐	☒	A description of all covariates tested
☐	☒	A description of any assumptions or corrections, such as tests of normality and adjustment for multiple comparisons
☐	☒	A full description of the statistical parameters including central tendency (e.g. means) or other basic estimates (e.g. regression coefficient) AND variation (e.g. standard deviation) or associated estimates of uncertainty (e.g. confidence intervals)
☐	☒	For null hypothesis testing, the test statistic (e.g. F, t, r) with confidence intervals, effect sizes, degrees of freedom and P value noted<br>*Give P values as exact values whenever suitable.*
☒	☐	For Bayesian analysis, information on the choice of priors and Markov chain Monte Carlo settings
☒	☐	For hierarchical and complex designs, identification of the appropriate level for tests and full reporting of outcomes
☐	☒	Estimates of effect sizes (e.g. Cohen's d, Pearson's r), indicating how they were calculated

Our web collection on statistics for biologists contains articles on many of the points above.

Software and code

Policy information about availability of computer code

Data collection	Visual stimuli were presented using PsychToolBox (version 3.0.13).
Data analysis	We used MATLAB (version 2016b) and PyTorch (version 0.4.1) for all data analyses.

For manuscripts utilizing custom algorithms or software that are central to the research but not yet described in published literature, software must be made available to editors and reviewers. We strongly encourage code deposition in a community repository (e.g. GitHub). See the Nature Portfolio guidelines for submitting code & software for further information.

Data

Policy information about availability of data

All manuscripts must include a data availability statement. This statement should provide the following information, where applicable:

- Accession codes, unique identifiers, or web links for publicly available datasets
- A description of any restrictions on data availability
- For clinical datasets or third party data, please ensure that the statement adheres to our policy

The data that support the findings of this study are available in the following repository: https://doi.org/10.17605/OSF.IO/VF2XK. Source data for all figures in the paper are also provided.

Field-specific reporting

Please select the one below that is the best fit for your research. If you are not sure, read the appropriate sections before making your selection.

- [x] Life sciences
- [ ] Behavioural & social sciences
- [ ] Ecological, evolutionary & environmental sciences

For a reference copy of the document with all sections, see nature.com/documents/nr-reporting-summary-flat.pdf

Life sciences study design

All studies must disclose on these points even when the disclosure is negative.

Sample size	To determine sample size and optimize experimental design, we performed a series of statistical simulations in advance in which we varied the magnitude of the hypothesized straightening and entangling effects (effect size estimates based on model simulations in Henaff et al., 2019), the number of simultaneously recorded units, the amount of shared response variability, and the number of repeated trials. These simulations informed our experimental design and sample size choice (Aim: 5-10 populations, >10 units per population; Outcome: 7 populations, 40 units/population on average).
Data exclusions	We excluded 2 recordings (out of 9) as their recording was incomplete. We analyzed neural trajectories from the remaining 7 recordings, and excluded trajectories when our analysis could not account for the mean, variance, and covariance of firing rates. Specifically, we analyzed trajectories when the explained variance for the mean, variance, and covariance of firing rates exceeded 75%, 50%, and 25% respectively. In addition, we excluded trajectories if the mean distance of the trajectory was noticeably low. Overall, we excluded 25% of all trajectories from the 7 recordings. We assessed the impact of these criteria on our conclusions (Supplementary Figure 8), and found it to be negligible.
Replication	We replicated neural straightening effects while varying the spatial and temporal resolution of movies (Figure 6).
Randomization	In all analyzed experiments (image sequences, oriented gratings, sparse noise), stimuli were presented in a randomized order.
Blinding	Stimuli from natural and unnatural conditions were randomly interleaved throughout the experiment, making blinding unnecessary.

Reporting for specific materials, systems and methods

We require information from authors about some types of materials, experimental systems and methods used in many studies. Here, indicate whether each material, system or method listed is relevant to your study. If you are not sure if a list item applies to your research, read the appropriate section before selecting a response.

Materials & experimental systems

n/a	Involved in the study	
☒	☐	Antibodies
☒	☐	Eukaryotic cell lines
☒	☐	Palaeontology and archaeology
☐	☒	Animals and other organisms
☒	☐	Human research participants
☒	☐	Clinical data
☒	☐	Dual use research of concern

Methods

n/a	Involved in the study	
☒	☐	ChIP-seq
☒	☐	Flow cytometry
☒	☐	MRI-based neuroimaging

Animals and other organisms

Policy information about studies involving animals; ARRIVE guidelines recommended for reporting animal research

Laboratory animals	We collected extracellular voltage activity from four male cynomolgus monkeys (Macaca fascicularis) aged 4 to 8 years.
Wild animals	This study did not involve wild animals.
Field-collected samples	This study did not involve field-collected samples.
Ethics oversight	All experiments were conducted in compliance with the NIH Guide for the Care and Use of Laboratory Animals, and with approval of the University of Texas Institutional Animal Care and Use Committee.

Note that full information on the approval of the study protocol must also be provided in the manuscript.

March 2021
